# The Status of Health Promoting Lifestyle among Students of Tabriz, Northwestern Iran

**Published:** 2018-12

**Authors:** Jafar Sadegh TABRIZI, Najibeh KHOSHMARAM, Leila DOSHMANGIR, Elham SHAKIBAZADEH

**Affiliations:** 1. Tabriz Health Services Management Research Center, Tabriz University of Medical Sciences, Tabriz, Iran; 2. Dept. of Health Services Management, Tabriz University of Medical Sciences, Tabriz, Iran; 3. Dept. of Health Education and Promotion, School of Public Health, Tehran University of Medical Sciences, Tehran, Iran

## Dear Editor-in-Chief

Health is a “State of complete physical, mental, and social well-being, and not merely the absence of disease or infirmity” (1). “Health promotion is the process of enabling people to increase control over, and to improve their health” (2). Health promoting behaviors are formed during adolescences and often remain until adulthood. Adolescence is a transitional stage from childhood to adulthood and during this period, adolescents experience many changes. These include physical growth, new social relations and emotions. Adolescent population in Iran was 12 million in 2011 (16% of population) (3). Adolescences lifestyle has a significant effect on burden of diseases in the future (4). Behaviors such as smoking, unhealthy nutrition, risky sexual behaviors, and sedentary life style usually start in this period of life which can lead to chronic diseases in adulthood (5). Health-promoting lifestyle can be trained to students; and schools are pivotal settings in which health-promoting lifestyle can be educated (6).

We aimed to explore health-promoting lifestyle in health different dimensions among students living in Tabriz, Northwestern Iran. The health-promoting lifestyle in students was moderate. The maximum score belonged to the social health dimension (75.12± 14.14), and the minimum score belonged to the physical health dimension (64.39 ± 12.47). The mental health dimension score was lower than average. In addition, the physical and mental health dimensions scores among boys were more than girls. Moreover, the score of social health dimension for girls was more than boys. There was significant difference in health-promoting lifestyle score between the female and male participants. Pearson correlation test showed that there were significant correlations between social health dimension and age.

Regarding the low score of health-promoting behaviors, especially in dimension of physical health among students, health training programs toward increasing physical health dimension is recommended. With the increasing knowledge level of students on health-promoting behaviors, a behavior change would be observed.
